# Keel Fracture Causes Stress and Inflammatory Responses and Inhibits the Expression of the Orexin System in Laying Hens

**DOI:** 10.3390/ani9100804

**Published:** 2019-10-15

**Authors:** Haidong Wei, Chun Li, Hongwei Xin, Shuang Li, Yanju Bi, Xiang Li, Jianhong Li, Runxiang Zhang, Jun Bao

**Affiliations:** 1 College of Animal Science and Technology, Northeast Agricultural University, Harbin 150030, China; weihaidongneau@163.com (H.W.); yanju_bi@163.com (Y.B.); ryan626@139.com (X.L.); 2 College of life Science, Northeast Agricultural University, Harbin 150030, China; lichun0917123@163.com (C.L.); 15804639630@163.com (S.L.); jhlineau@126.com (J.L.); 3 Institute of Agriculture, The University of Tennessee, Knoxville, TN 37996-4506, USA; hxin2@tennessee.edu

**Keywords:** laying hens, keel fracture, stress, inflammation, orexin

## Abstract

**Simple Summary:**

Keel fracture is an important health and welfare problem in laying hens in all production systems. Previous studies have shown that keel fracture can influence hens’ behavior, reduce production performances, and cause pain in laying hens. Additionally, keel fracture also affects their feed intake. However, it is not clear whether the keel fracture induces stress, inflammation, or influences the orexin systems in laying hens. Orexin, also called hypocretin, is associated with the regulation of feed intake, energy homeostasis, and metabolism in poultry animals. Therefore, this study aimed to investigate the effects of keel fracture on stress and inflammatory responses and the activity of the orexin system of laying hens. Our results indicate that keel fracture not only induced stress and inflammatory responses, but inhibited the activity of the orexin system in laying hens. This study provides insights into the adverse effects of keel fracture on laying hens.

**Abstract:**

Keel fracture has negative effects on the health and welfare of laying hens. We investigated effects of keel fracture on stress, inflammation, and the orexin system in laying hens. Ninety 17-week-old Lohmann white laying hens were palpated and euthanatized at 42 weeks old, and marked as normal keel (NK)/fractured keel (FK) from absence/presence of keel fracture. Serum, brain, liver, and abdominal-muscle samples were collected from 10 NK and 10 FK hens to determine the stress and inflammatory responses and the activity of orexin systems by corticosterone content, expression of heat shock proteins (*TNF-α 60*, 70, 90), and inflammatory factors (tumor necrosis factor *(TNF)-α*, nuclear factor-kappa Bp65 (*NF-κBp65*), inducible nitric oxide synthase (*iNOS*), prostaglandin E synthases (*PTGEs*), cyclooxygenase-2 (*COX-2*), interleukin-1β (*IL-1β*)), orexin (*ORX*), and orexin-receptor 1/2 (*ORXR1/ORXR2*). The FK hens had higher serum corticosterone content, Hsps, and inflammatory factor mRNA expression levels than NK hens, although levels of *iNOS* in the liver and *TNF-α* in the muscle were similar. Protein levels of Hsp70 and Hsp90 in the brain and liver, iNOS and COX-2 in the liver, NF-κBp65, iNOS, and COX-2 in the brain of FK hens were increased compared with NK hens. Furthermore, FK hens had lower mRNA expression of *ORX*, *ORXR1*, and *ORXR2* than NK hens. Therefore, keel fracture causes stress and inflammation, and inhibits the expression of the orexin system in laying hens.

## 1. Introduction

Keel fracture is an important problem in welfare and health in modern production systems of laying hens. Several studies have investigated the percentage of occurrence of keel fracture in different housing systems, genetic lines, and ages of laying hens, and have reported its prevalence as 30–97% [1,2,3,4,5]. Keel fracture not only influences the behavior and welfare of laying hens, but also reduces their performance, egg, and bone quality [6,7,8,9]. In rat tibia and human femur models, fracture induces pain, stress, and inflammatory responses [10,11]; however, we do not know whether this response can be seen in laying hens with keel fracture.

Heat shock response and inflammatory response are the primary pathways to initiate the synthesis of some special heat shock proteins (Hsps) and inflammatory cytokines [12]. The Hsps are highly conserved molecular chaperones, and their expression levels are normally low under physiological conditions. However, these are rapidly synthesized under stress conditions, such as extreme temperatures and oxidative stress, to improve the cellular tolerance to damage induced by stress, maintain normal metabolic function, and increase cellular viability [13,14]. Studies have reported that acute heat and cold stress upregulate the expression of *Hsp60*, *Hsp70*, and *Hsp90* [15,16]. Additionally, the inflammatory response induced by fracture occurs mainly in the initial stage of healing, and plays a pivotal role in early repair [17]. Inflammation cytokines, such as tumor necrosis factor-alpha (*TNF-α*), nuclear factor-kappa B (*NF-κB*), cyclooxygenase-2 (*COX-2*), prostaglandin E synthases (*PTGEs*), and interleukin-1β (*IL-1β*), are produced at the fracture site. Their players are involved in the regulation of inflammatory response, renewing and differentiation of mesenchymal progenitor cells, as well as in the promotion of fracture healing [18,19,20]. Therefore, Hsps and inflammatory cytokines play a vital role in the recovery of tissue damage.

Orexin (*ORX*), also known as hypocretin, has two molecular forms, orexin-A and orexin-B [21], which are derived from a common prepro-orexin by proteolytic cleavage. Orexins bind to two widely expressed G-protein-coupled receptors, called orexin receptor 1 (*ORXR1*) and orexin receptor 2 (ORXR2) [22]. *ORX* and *ORX* receptors are abundantly expressed in the brain, hypothalamus, liver, muscle, and intestines of birds [15]. Many studies have indicated that *ORX* regulates the sleep/wake cycle, reward-seeking behavior, and energy homeostasis in mammals [23,24]. In avian species, *ORX* also regulates the mitochondrial biological function [25] and metabolism [26]. Greene et al. [27] and Nguyen et al. [15] found that heat stress and oxidative stress downregulated the expression of *ORX* and its receptor genes in the liver and muscle tissues of birds. This suggests that *ORX* is involved in stress response and regulation of the physiological process. As laying hens with keel fracture have higher feed consumption [7], it may be associated with the altered expression levels of *ORX* and its receptors.

Therefore, to investigate the negative effect of keel fracture on laying hens, the expression levels of Hsps, inflammatory factors, and *ORX* and its receptors were evaluated in this study. We hypothesized that keel fracture can cause stress and inflammation and inhibit the activity of orexin system in laying hens.

## 2. Materials and Methods

### 2.1. Animals and Management

All experiments were approved by, and conducted according to, the guidelines of the Institutional Animal Care and Use Committee of Northeast Agriculture University (Ethic code: IACUCNEAU20150616). Ninety 17-week-old healthy Lohmann white laying hens with normal keel bone were used in the current study. They were individually housed in furnished cages, which were placed in the semi-enclosed house with a combination of natural and artificial light. The artificial light program included 16 h light (5:00–21:00) and 8 h dark, and light intensity was 18–22 lux. Each cage was of 50 cm length × 70 cm width × 70 cm height, with a closed nest box, two wooden square perches, one nipple drinker, and one rectangular feeder. During the entire experiment period (17–42 weeks of age), all laying hens had free access to feed and water, the temperature of the house was 20–28 °C, and the relative humidity was 45–70%. Laying hens were provided with a standard commercial layer diet, mainly with metabolic energy of 2800 kcal/kg, and crude protein 16.08%.

### 2.2. Assessment of Keel Fracture

For the assessment of keel condition, we followed the method by Scholz et al. [28] and Casey-Trott et al. [29]. In brief, all laying hens were palpated at 42 weeks of age (WOA) to evaluate the keel bone status that was mainly divided into three categories: Normal keel (NK), deviated keel (DK), and fractured keel (FK). If both deviation and fracture existed, the hen was considered to have FK, as fracture presumably induced more pain than deviation [28]. After palpation, the hens were euthanized and dissected. Using visual observation, keel bone status of each was confirmed and marked as NK or FK based on the absence or presence of keel fracture, respectively. There were 49 hens with FK, 22 hens with DK, and 19 hens with NK.

### 2.3. Sample Collection

After euthanasia and keel bone validation, the blood of NK and FK hens (n = 10 each) was collected, and centrifuged at 2500× *g* for 15 min. The serum thus obtained was stored at −20 °C for the determination of corticosterone content. The brain, liver, and abdominal muscle tissues from NK and FK hens were collected and stored at −80 °C for further use.

### 2.4. Determination of Serum Corticosterone Content

The concentrations of serum corticosterone from NK and FK hens were measured in triplicate using a commercially available ELISA kit (Shanghai Jinma Laboratory Equipment Corporation., Ltd., Shanghai, China) according to the manufacturer’s instructions. Optical densities (OD) were measured at 450 nm by an ELISA reader (Biotek Instrument Inc. Winooski, VT, USA).

### 2.5. Total RNA Extraction, Reverse Transcription, and Quantitative Real-Time PCR (qRT-PCR) Analysis

Total RNA was extracted from each frozen brain, liver, and abdominal muscle sample using an RNAiso Plus Kit, following the manufacturer’s instructions (Takara, Dalian, China). The concentration and purity of the total RNA in each sample was determined at 260 nm and 260/280 nm (Gene Quant 1300/100, Los Angeles, CA, USA), respectively. The reverse transcription process was performed using oligo dT and Superscript II reverse transcriptase according to the RR047 kit manufacturer’s protocol (Takara, Dalian, China), and the cDNAs were diluted using sterile water and stored at −80 °C for qRT-PCR analysis.

Special forward and reverse primer sequences for *Hsp60*, *Hsp70*, *Hsp90*, *TNF-α*, *COX-2*, *PTGEs*, *NF-κBp65*, inducible nitric oxide synthase (*iNOS*), *IL-1β*, and glyceraldehyde 3-phosphate dehydrogenase (*GAPDH*) genes were designed using Primer Premier Software 5.0 (PREMIER Biosoft International, Palo Alto, CA, USA). The primer sequences for each gene were as listed in Table 1, and the primer sequences of *ORX*, *ORXR1*, and *ORXR2* were referenced as per the published article [15]. The qRT-PCR was performed using Light Cycler^®^ 480 qPCR system (Roche, Rotkreuz, Switzerland) and reaction mixture system was 10 µL, including 1 mL diluted cDNA, 0.3 mL of each primer (10 mM), 5 mL 2× Roche Fast Universal SYBR Green Master kit (Roche, Mannheim, Germany), and 3.4 mL PCR grade water. The qPCR conditions were: Initial heating to 50 °C for 2 min and pre-denaturation at 95 °C for 10 min, followed by 40 cycles at 95 °C for 15 s and 60 °C for 1 min. The qRT-PCR reaction was performed in triplicate for each sample, and the threshold cycle (Ct) value was calculated by a mean value of triplicate. The relative expression of each gene was calculated according to the 2^−ΔΔ*C*t^ method of Schmittgen and Livak [30], and the housekeeping gene *GAPDH* was used as the internal reference to normalize the expression of other genes.

### 2.6. Western Blotting

Frozen brain and liver tissues (100 mg each) were separately put in the lysis buffer containing 1 mM PMSF (Beyotime, Shanghai, China) to homogenize at low temperature. Homogenates were centrifuged at 12,000× *g* at 4 °C for 5 min to collect the supernatants, which were quantified using the Enhanced BCA Protein Assay Kit (Biosharp, Beijing, China) and dissolved with 5× sample loading buffer. Equal amounts of total protein (40 µg) were subjected to 10% sodium dodecyl sulphate–polyacrylamide gel electrophoresis (SDS-PAGE) gels. The separated proteins were transferred onto nitrocellulose membranes using a DYCP-40C semi-dry transfer apparatus (LIUYI, Beijing, China). The membranes were blocked with 5% skim milk at room temperature for 1 h, and then incubated overnight at 4 °C with primary antibodies against Hsp40 (1:3000), Hsp70 (1:5000), and Hsp90 (1:3000), which three antibodies were supplied by Professor Shiwen Xu, Northeast Agricultural University, Harbin, China. The primary antibodies of NF-κBp65 (1:300, Santa Cruz, CA, USA), iNOS (1:500, Proteintech, Chicago, IL, USA), COX-2 (1:500, Wanleibio, Shenyang, China), and β-actin (1:7000, Abcam, Cambridge, UK) were also incubated overnight at 4 °C. The horseradish peroxidase-conjugated goat anti-rabbit IgG (1:40,000, Bioss Antibodies, Beijing, China) was used as the secondary antibody to localize the primary antibodies. The signal was measured by an enhanced chemiluminescence detection kit (Biosharp, Beijing, China) and protein bands were detected by a gray scale scanner (GeneGnome XRQ; Syngene Corp., Cambridge, UK). Finally, protein band intensities were analyzed by Image J software, and the relative abundance of the protein was expressed as the ratio of optical density of each protein to that of β-actin.

### 2.7. Statistical Analysis

Statistical analyses were carried out using SPSS 22 for Windows (SPSS Inc., Chicago, IL, USA). All data were tested for normal distribution using the Kolmogorov–Smirnov test, and then an independent *t*-test was used to compare the mean of each indicator from NK and FK hens. The results are presented as the mean ± SEM, and *p* ≤ 0.05 was considered as a statistically significant difference.

## 3. Results

### 3.1. Determination of Serum Corticosterone Concentration

**Figure 1:**
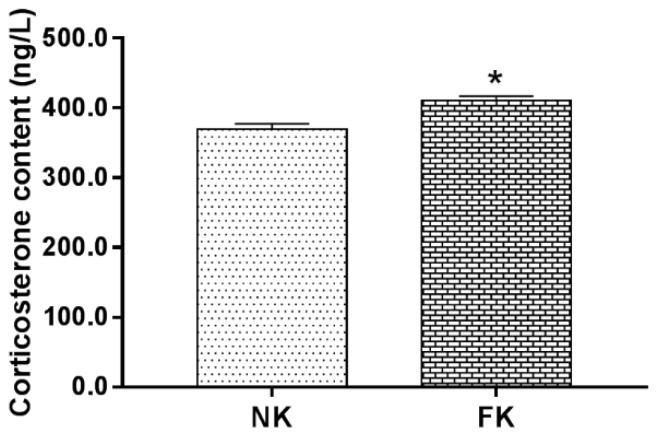
The concentration of serum corticosterone of laying hens with normal keel (NK) or fractured keel (FK). * *p* ≤ 0.05.

As shown in Figure 1, the serum corticosterone concentration in the FK hens was significantly higher than that of the NK hens (*p* < 0.05) at 42 WOA.

### 3.2. Expression Levels of Hsps in Hen Brain, Liver, and Muscle Tissues

**Figure 2:**
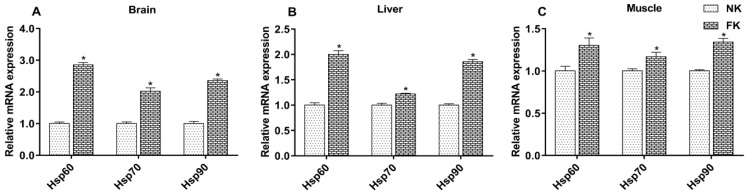
Expression levels of *Hsp60*, *Hsp70*, and *Hsp90* mRNA in the brains (**A**), livers (**B**), and muscles (**C**) of laying hens with normal keel (NK) or fractured keel (FK). * *p* ≤ 0.05.

The relative expression levels of *Hsp60*, *Hsp70*, and *Hsp90* mRNA in the brains, livers, and muscles of NK and FK hens are shown in Figure 2. At 42 WOA, the mRNA expression levels of these Hsps in the brain, liver, and muscle tissues of FK hens were significantly higher than that of NK hens (*p* < 0.05).

**Figure 3:**
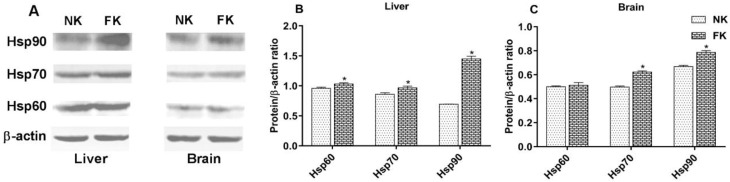
Protein bands of Hsps and β-actin (**A**), and protein expression of Hsp60, Hsp70, and Hsp90 in the livers (**B**) and brains (**C**) of laying hens with normal keel (NK) or fractured keel (FK). * *p* ≤ 0.05.

Protein levels of Hsp60, Hsp70, and Hsp90 in the brains and livers of NK and FK hens are shown in Figure 3. Similar to the mRNA results, compared to NK hens, the protein expression of Hsp60 in the livers, and Hsp70 and Hsp90 in the livers and brains of FK hens were significantly increased (*p* < 0.05), although no significant difference was detected in the levels of Hsp60 in the brains (*p* > 0.05).

### 3.3. Expression Levels of Inflammatory Factors in Hen Brains, Livers, and Muscles

**Figure 4:**
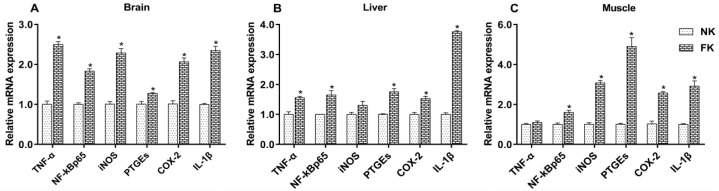
Expression levels of *TNF-α*, *NF-κBp65*, *iNOS*, *PTGEs*, *COX-2*, and *IL-1β* mRNA in the brains (**A**), livers (**B**), and muscles (**C**) of laying hens with normal keel (NK) or fractured keel (FK). * *p* ≤ 0.05.

The relative expression levels of mRNA for *TNF-α*, *NF-κBp65*, *iNOS*, *PTGEs*, *COX-2*, and *IL-1β* in the brain, liver, and abdominal muscle of NK and FK hens are shown in Figure 4. The expression of all the evaluated inflammatory factors was significantly higher in the brains of FK hens than those of NK hens (*p* < 0.05). Compared to NK hens, the expression levels of *TNF-α*, *NF-κBp65*, *PTGEs*, *COX-2*, and *IL-1β* mRNA in the livers, and *NF-κBp65*, *iNOS*, *PTGEs*, *COX-2*, and *IL-1β* mRNA in the abdominal muscles of FK hens was significantly increased (*p* < 0.05). No difference was observed in mRNA expression of iNOS in the livers or *TNF-α* in the abdominal muscles between the NK and FK hens (*p* > 0.05). 

**Figure 5:**
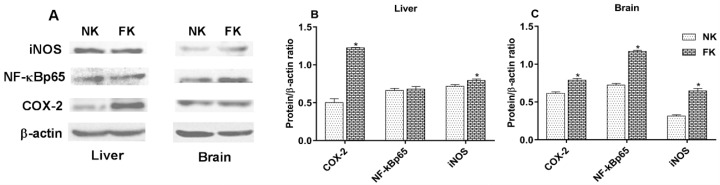
Protein bands of inflammatory factors and β-actin (**A**), and protein levels of COX-2, NF-κBp65, and iNOS in the livers (**B**) and brains (**C**) of laying hens with normal keel (NK) or fractured keel (FK). * *p* ≤ 0.05.

Protein expression levels of COX-2, NF-κBp65, and iNOS in the livers and brains are shown in Figure 5. The protein levels of COX-2 and iNOS in the livers, and COX-2, NF-κBp65 and iNOS in the brains of FK hens significantly increased (*p* < 0.05). However, there was no difference (*p* > 0.05) in NF-κBp65 in the livers of FK hens compared to NK hens.

### 3.4. Expression Levels of Orexin and Orexin Receptors in Hen Brains, Livers, and Muscles

**Figure 6:**
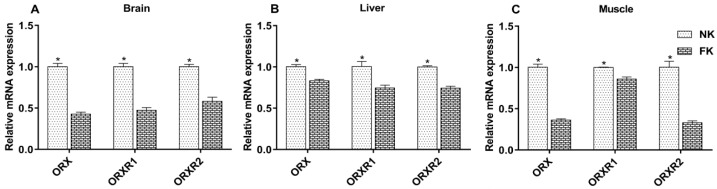
Expression of *ORX*, *ORXR1*, and *ORXR2* mRNA in the brains (**A**), livers (**B**), and muscles (**C**) of laying hens with normal keel (NK) or fractured keel (FK). * *p* ≤ 0.05.

As shown in Figure 6, at 42 WOA, compared to NK hens, the FK hens showed a significant decrease in relative mRNA expression of *ORX*, *ORXR1*, and *ORXR2* in the brain, liver, and abdominal muscle (*p* < 0.05).

## 4. Discussion

In modern housing systems, laying hens still face considerable welfare and health challenges, and one of which is keel fracture. Keel fracture not only influences the behavior, welfare, and production performance of hens [6,7,31], but also causes physiological stress [32]. When animals are exposed to emotional, physiological, or environmental stress, different biological response systems are activated to deal with the potential threat of such stress [33]. On the one hand, these responses would induce changes in behaviors and immune functions; on the other hand, they could alter the autonomic nervous system and neuroendocrine system (hypothalamic–pituitary–adrenal, HPA) [34]. In poultry species, corticosterone is a vital product at the end of the HPA axis [35]. Therefore, the quantitative determination of corticosterone level has become an important indicator to evaluate the stress levels in the birds [36,37]. Some reports suggested that serum corticosterone content of chickens was evidently elevated under stress conditions such as crowded stocking density and heat and cold stress; and when stress was eliminated, the corticosterone content returned to its prior stress (normal) level [37,38]. In this study, serum corticosterone content of FK hens was significantly higher than that of NK hens, indicating that keel fracture causes stress in laying hens.

The Hsps are a group of important molecular chaperones and involved in many cellular metabolic processes. They play a vital role in the folding/unfolding of proteins and the assembly/disassembly of protein complexes [39]. The *Hsp60* exists in the cytoplasm and mitochondrial matrix at normal conditions, and it can rapidly transfer from the cytoplasm to the mitochondrial matrix to repair the denatured proteins when the body is exposed to stress [40]. The expression of *Hsp70* is most abundant under stress conditions [41]. The *Hsp90* plays an important role in stress protection, mainly responsible for protein transport and degradation and maintaining the configuration of intracellular proteins [42]. There are reports that show that cold stress and oxidative stress upregulate the mRNA expression of *Hsp60*, *Hsp70*, and *Hsp90* in poultry animals [16,43]. Similar to the previous studies, our results found that keel fracture increased *Hsp60*, *Hsp70*, and *Hsp90* expression in the brain, liver, and muscle tissues of laying hens, indicating that the keel fracture causes stress. Additionally, as important molecular chaperones, Hsps also regulate bone metabolism and remolding. Wang et al. [44] reported that an elevated level of *Hsp60* can protect skeletal tissue by improving osteoblast activity against bone damage and the loss of trabecular microstructure in rats. *Hsp70* has stimulatory effects on bone morphogenetic protein secretion and increases the expression of these proteins in bone healing [45]. *Hsp90* inhibitor stimulates osteoclast activity and bone metastasis of human [46]. Therefore, increased expression of *Hsp60*, *Hsp70*, and *Hsp90* in FK hens may play a promoting effect on bone healing in this study.

The fracture causes physiological stress, which increases the levels of stress hormones and pain, as well as an inflammatory response [10,11]. Fracture healing mainly consists of three important phases in human and animals, each of which causes changes in bone structure and tissue formulation [47]. The initial stage of fracture repair with inflammation leads to the formation of hematoma and granulation tissue. Next, the formation of soft callus (including cartilaginous or chondroid tissue) at the fracture site occurs. Finally, the soft callus ossifies to form bony or hard callus tissue [17,47]. Hematoma that occurs in initial fracture repair is due to the infiltration of inflammatory factors and secretion of cytokines and growth factors [47]. In the mouse model, *TNF-α*, *IL-1*, and *IL-6* were produced at the fracture site within 24 h of the fracture, and were mainly involved in the renewal and differentiation of mesenchymal progenitor cells, and promoted the healing [18,48]. Otherwise, previous studies found that the expression of some inflammatory molecules such as *IL-1β*, *TNF-α*, *COX-2*, and *PTGEs* in mice and rats was increased during the osteogenic phases of the fracture repair [19,20]. In the present study, the expression of *TNF-α*, *NF-κBp65*, *iNOS*, *PTGEs*, *COX-2*, and *IL-1β* increased in the brain, liver, and abdominal muscle of FK hens. This indicates that keel fracture caused an inflammatory response in the hens, which in turn led to the production of inflammatory cytokines in large quantities at the fracture site. Furthermore, the high expression of these inflammatory cytokines may be related to bone repair and remodeling at the fracture site.

Orexin and its receptor genes are expressed in the brain, liver, and muscle tissues of poultry animals [15,49]. Many studies have found that *ORX* has multiple biological functions. It regulates sleeping, wakefulness, feeding behavior, and energy metabolism [23,24], as well as mediates emotions related to various stress in mammals [50,51]. Additionally, *ORX* regulates energy metabolism and feeding behavior in avian species [49]. Greene et al. [27] showed that heat stress and oxidative stress downregulated the expression of *ORX*, *ORXR1*, and *ORXR2* in the liver and muscle of quails, which suggests that orexin system plays an important role in avian response to stress. Research has also reported that the decreased expression of ORX-related genes was related to increased expression of *Hsp70* under heat stress; namely, the expression of the orexin system was mediated through *Hsp70* [15]. Therefore, on the one hand, the mRNA expression levels of *ORX*, *ORXR1*, and *ORXR2* in FK hens might be associated with high expression levels of Hsps (*Hsp60*, *Hsp70*, and *Hsp90*) in our study. On the other hand, Nasr et al. [7] found that laying hens with FK eat more than normal hens. We also speculate that keel fracture as an acute or a chronic stress factor, which can directly or indirectly inhibit the expression of *ORX*, *ORXR1*, and *ORXR2*, reduces the activity of the orexin system and then influences energy metabolism and feed consumption. Thus, the molecular mechanism of the fracture on the orexin system needs to be explored in future studies.

## 5. Conclusions

The present study indicates that keel fracture not only causes stress and inflammatory responses by increasing serum corticosterone content and upregulating the expression levels of Hsps and inflammatory factors, but also inhibits the expression of orexin and orexin receptor genes in laying hens.

## Figures and Tables

**Table 1:** Primer sequences for quantitative real-time PCR.

Gene	Reference Sequence	Primer Sequences (5′-3′)
*Hsp60*	NM_001012916.1	Forward: AGCCAAAGGGCAGAAATG Reverse: TACAGCAACAACCTGAAGACC
*Hsp70*	NM_001006685.1	Forward: CGGGCAAGTTTGACCTAA Reverse: TTGGCTCCCACCCTATCTCT
*Hsp90*	NM_001109785.1	Forward: TCCTGTCCTGGCTTTAGTTT Reverse: AGGTGGCATCTCCTCGGT
*TNF-α*	NM_204267	Forward: GCCCTTCCTGTAACCAGATG Reverse: ACACGACAGCCAAGTCAACG
*COX-2*	NM_001167718	Forward: TGTCCTTTCACTGCTTTCCAT Reverse: TTCCATTGCTGTGTTTGAGGT
*PTGEs*	NM_001194983.1	Forward: GTTCCTGTCATTCGCCTTCTAC Reverse: CGCATCCTCTGGGTTAGCA
*NF-κBp65*	NM_205134	Forward: TCAACGCAGGACCTAAAGACAT Reverse: GCAGATAGCCAAGTTCAGGATG
*iNOS*	NM_204961.1	Forward: CCTGGAGGTCCTGGAAGAGT Reverse: CCTGGGTTTCAGAAGTGGC
*IL-1β*	NM_204524.1	Forward: ACTGGGCATCAAGGGCTACA Reverse: GCTGTCCAGGCGGTAGAAGA
*ORX*	AB056748	Forward: CCAGGAGCACGCTGAGAAG Reverse: CCCATCTCAGTAAAAGCTCTTTGC
*ORXR1*	NM_205505	Forward: TGCGCTACCTCTGGAAGGA Reverse: GCGATCAGCGCCCATTC
*ORXR2*	AF408407	Forward: AAGTGCTGAAGCAACCATTGC Reverse: AAGGCCACACTCTCCCTTCTG
*GAPDH*	NM_204305.1	Forward: GCACGCCATCACTATCTTCC Reverse: CATCCACCGTCTTCTGTGTG
